# A low-dose therapy of fibrinogen supplement during perioperative period of total knee arthroplasty in an asymptomatic man with congenital dysfibrinogenemia: A case report

**DOI:** 10.1097/MD.0000000000031644

**Published:** 2022-11-18

**Authors:** Decheng Meng, Runzi Zhang, Chenni Ji, Shijun Gao, Juan Wang

**Affiliations:** a Department of Joint Surgery, The Third Hospital of Hebei Medical University, Shijiazhuang, Hebei, China; b NHC Key Laboratory of Intelligent Orthopedic Equipment (The Third Hospital of Hebei Medical University), Shijiazhuang, Hebei, China.

**Keywords:** congenital dysfibrinogenemia, knee osteoarthritis, total knee arthroplasty

## Abstract

Congenital dysfibrinogenemia (CD) is a rare coagulation system disease that is often treated without unified management. Individualized treatment thereof presents clinicians with great challenges.

**Patient concerns:**

A patient who was about to undergo total knee arthroplasty was found to have CD.

**Diagnoses:**

Coagulation screening revealed low fibrinogen, prolonged thrombin time, minor prolonged prothrombin time, and normal activated partial thromboplastin time were detected during admission, but no abnormal personal and family history findings were observed. Therefore, CD and hypofibrinogenemia were suspected. The gene sequencing confirmed the diagnosis of CD.

**Interventions:**

The patient received plenty and low level of fibrinogen concentrate during 2 perioperative periods, respectively.

**Outcomes:**

Successful clinical outcomes were obtained using different treatment strategies.

**Lessons:**

In contrast to prior case reports, this case illustrates the feasibility of low dosing of fibrinogen supplements within an asymptomatic patient in a selective operation. Changes in the level of fibrinogen and fibrin degradation product are of great importance for individualized treatment after supplementation.

## 1. Introduction

Congenital dysfibrinogenemia (CD), a rare autosomal dominant inheritance disease, manifests heterogeneous clinical features, making it impossible to estimate the prevalence of CD, like congenital afibrinogenemia, and there is no correlation between the genotype and the phenotype.^[1–3]^ Among heterogeneous clinical symptoms, the bleeding phenotype is more frequent than thrombosis and asymptomatic, but the phenotype at diagnosis can only represent situation at the moment.^[4,5]^ In supplement therapy, different variants respond differently to fibrinogen concentrate (FC).^[6]^ Therefore, it is difficult to establish a safe and effective enhanced recovery after surgery protocol during the perioperative period of total knee arthroplasty. In such rare patients, multidisciplinary collaboration is necessary, with an urgent need for complying with total knee arthroplasty (TKA) guidelines to confront a rapidly aging society.

We reported the case of a patient with CD with severe knee arthropathy who was treated with primary staged bilateral TKAs. His case obtained similarly successful outcomes through 2 different doses of FC. The patient provided written informed consent for this report.

## 2. Case report

The 74-year-old male patient was diagnosed with bilateral severe knee osteoarthritis (Kellgren-Lawrence grade 4) according to full-length weight-bearing anterior-posterior, lateral, Rosenberg and patella skyline radiographs. He was admitted to the joint surgery department for arthroplasty of the right knee. His personal history suggested no comorbidity, trauma history or surgical history. Preoperative global coagulation screening revealed low fibrinogen using the Clauss assay, with prolonged thrombin time, minor prolonged prothrombin time and normal activated partial thromboplastin time. No abnormality was found in other preoperative examinations. A family history review showed that the patient’s older son had low fibrinogen discovered just before his renal surgery 1 year prior. Preoperative fibrinogen replacement therapy was given, and no postoperative bleeding or thrombosis complication developed. Both of the patient’s 2 daughters underwent knee surgery, and no coagulation abnormalities were found. In addition, the patient’s second son attended to our outpatient department for coagulation function tests, and his results also revealed a low fibrinogen level (0.27 g/L). No family members have ever experienced a bleeding or thrombotic event. Consequently, the patient was suspected of having congenital hypofibrinogenemia (CH) or dysfibrinogenemia.

**Figure 1.**
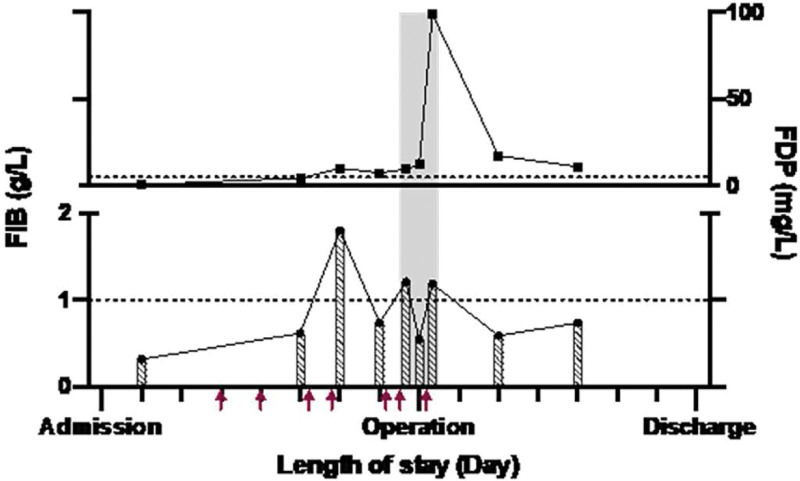
Changes of FIB and FDP during hospitalization and the management of low fibrinogen. FIB, fibrinogen activity level; FDP, fibrinogen degradation product (normal range, 0–5 mg/L); Gray column, the operation day, that is, the 9th day of hospitalization; Red arrow, infusion of 2g human fibrinogen concentrate.

Unfortunately, the family refused any other blood test for further diagnosis. To maximize perioperative safety, fibrinogen replacement therapy with an intravenous infusion of FC was included in preoperative preparation, aiming at a fibrinogen level of 1.0 to 1.5 g/L. However, fibrinogen degradation increased, and fibrinogen levels decreased quickly once they reached the target range. This manifestation made us more inclined to assume the existence of inactive variants but able to trigger hyperfibrinolysis. Therefore, the treatment strategy was adjusted (Fig. 1). TKA procedure was performed on day 9, even though the level of fibrinogen was only 0.55 g/L just before surgery. The procedure lasted 1.5 hours, and a dose of FC was supplemented intraoperatively. The blood loss after releasing the tourniquet was as usual, with a total amount of 300 mL. The postoperative management followed our enhanced recovery after surgery protocol, including the intravenous infusion of tranexamic acid (TXA, 1 g Bid, 3 days), stepped analgesia, prophylactic doses of low molecular weight heparin (LMWH, 6000 iu, q24h) started 24 hours postoperatively, mobilization via a walker started on a postoperative day 1, ankle pump exercise, active and passive ranges of motion exercises, and muscle-strengthening exercises. Deep vein thrombosis (DVT) involving the intramuscular calf vein and the peroneal vein of the operated leg was detected on postoperative day 4, and then therapeutic doses of LMWH (6000 iu, q12h) were administered for treatment. Recanalization of the DVT was shown by ultrasound 3 days later. However, a reduced platelet count (41.4 × 10^9^/L) was discovered on postoperative day 2, presumably a side effect of LMWH. Otherwise, the patient recovered without delayed hemorrhage, periarticular hematoma, pulmonary embolism, or early infection.

**Figure 2.**
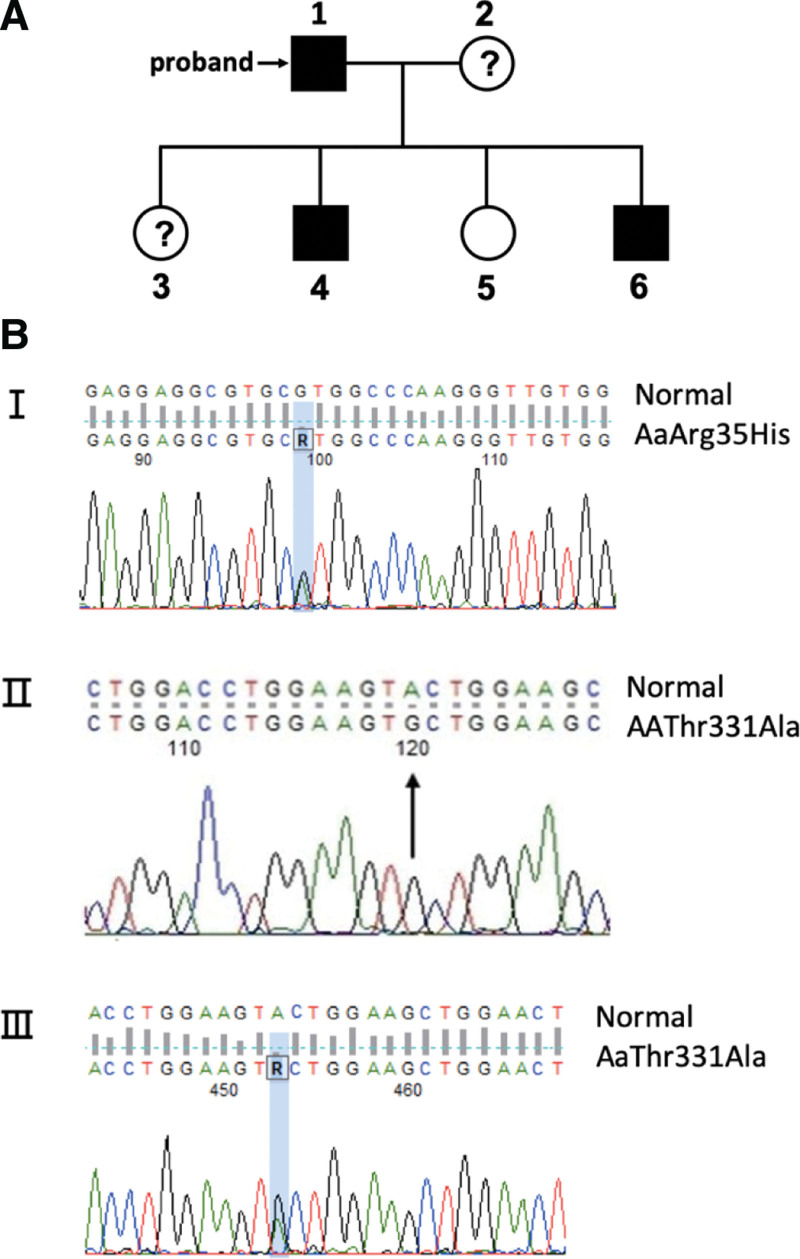
The results of FG gene sequencing and coagulation tests of the family members. (a) The pedigree of the family. The patient’s wife (2), older daughter (3) and older son (4) did not participate the research. However, the older daughter did not show abnormality in fibrinogen when she received knee surgery previously, and the older son showed lower level of fibrinogen when he received renal surgery; (b) The missense mutations found in the 3 tested family members. I, heterozygous p.Arg35His in FGA exon 2; II, homozygous p.Thr331Ala in FGA exon 5; III, heterozygous p.Thr331Ala in FGA exon 5. FC = fibrinogen concentrate.

During the follow-up 3 months after surgery, the patient exhibited excellent right knee function, without DVT of both low extremities. Total joint replacement of the left knee was scheduled as per his request. Upon admission, his level of fibrinogen activity was 0.66 g/L. After signing the consent form for this research, the patient, his second son and second daughter all received a fibrinogen antigen test via immunoturbidimetry and FG gene sequencing by a polymerase chain reaction method in addition to the routine coagulation function screening (Fig. 2 and Table 1). The missense mutation of EXON 2 on FGA gene, which was found in both the patient and his son, presumably is the pathological mutation of the hereditary abnormal fibrinogen disease in this family. Other mutations amongst the family members may be clinically irrelevant. Based on the laboratory results and the family’s pedigree, the patient’s diagnosis was modified to CD.

**Table 1:** Bleeding screening and gene sequencing.

Family member	FIB (activity) 2.0–4.0 g/L	FIB (antigen) 2.0–4.0 g/L	PT 10–14s	APTT 23.7–36.0s	TT 13.3–19.3s	FG gene mutation(s)
1	0.32	2.72	14.4	29.8	37.7	I, II
5	3.68	3.27	11.0	28.5	15.8	III
6	0.28	2.43	14.8	25.4	34.6	I, III

APTT = activated partial thromboplastin time, FIB = fibrinogen, PT= prothrombin time, TT = thrombin time.

Based on the patient’s benign course during and after his previous right knee surgery, the perioperative management at this time was carried out following our routine protocols without pre- and postoperative fibrinogen supplements. The left knee replacement was performed with the intraoperative infusion of 2 g FC. The intraoperative blood loss amounted to 400 mL. There was no complication such as severe pain, delayed hemorrhage, periarticular hematoma, DVT, or early infection, and the patient was discharged with a range of motion from full extension to 105° flexion on postoperative day 4. At a 1-month follow-up, the patient reported mild pain (visual analog score of 2) and good function in activity of daily living scale. By 3 months, the range of motion had plateaued from full extension to 120° flexion. During the year since the patient underwent TKA, his knee function recovered well without any bleeding and thrombotic events and the Forgotten Joint Score was 85.

## 3. Discussion

This case report not only is the first reported case of severe knee arthropathy in a patient with dysfibrinogenemia but also appears to be the first report of a patient with low dosing of fibrinogen supplement during the perioperative period.

Mutation of the fibrinogen alpha chain gene (c.104G > A) was revealed using gene sequencing in our patient, which is 1 of the hotspot mutations.^[7]^ However, minimal data describe how patients with this mutation are treated during the perioperative period, and major schemes are provided based on CH.^[4]^ We suggest that the relationship between variants and phenotypes must be further researched; however, the absence of an accurate relationship makes it impossible to directly assess the risk of bleeding and thrombosis. In our patient, DVT involving the intramuscular calf vein and the peroneal vein of the operated leg occurred during the first perioperative period but did not occur during the second perioperative period. Since these types of thrombosis also frequently occur in patients without CD after TKA, it does not indicate a relationship between the thrombotic phenotype, variants, and treatment strategy.

Many previous studies have reported the heterogeneous phenotypes of CD. Of these, the incidence of bleeding and thrombosis was observed in 34% and 9%, respectively, in the study conducted by Shapiro et al,^[8]^ 57% and 24%, respectively, in the study undertaken by Miesbach et al,^[9]^ 48% and 27%, respectively, in the study conducted by Casini et al,^[3]^ and 27% and 4%, respectively, in the study conducted by Zhou et al^[10]^ Those heterogeneous phenotypes highlight the challenges in treating CD patients, especially when asymptomatic patients experience complications. Current therapeutic schedules are driven by expert opinion and recommend individualized treatment in CD patients.^[4,11]^ Since asymptomatic pregnant women are more likely to be diagnosed because of indispensable pregnancy tests, there are recommendations for managing pregnancy in CD patients.^[12]^ However in a selective operation, most therapeutic schedules refer to CH patients.

The asymptomatic phenotype indirectly proved that some variants have unknown coagulation functions. This case manifests the challenges of fibrinogen supplements during a selective operation. The phenomenon of actual lower than expected fibrinogen activity has been found in previous studies according to subtle changes in fibrinogen activity.^[12,13]^ The observation has supported that dysfunctional fibrinogen may interfere with the function of replacing fibrinogen.^[6]^ As this case shows, during the first course, changes in the level of fibrinogen and FDP would suggest hyperfibrinolysis given the management of low fibrinogen. Contrary to previous studies, we then applied a low dose of fibrinogen supplement to undertake perioperative coagulation management and obtained a successful outcome during the second course. Although we agree with the aforementioned therapeutic schedules, the target value of more than 1 or 1.5 g/L may be difficult to achieve or maintain. We suggest caution in basing future treatment upon case-controlled trials to explore whether a lower-dose fibrinogen supplement is appropriate for treating asymptomatic CD patients. Within the aging society, an increasing number of patients with knee osteoarthritis need surgery; hence, more asymptomatic CD patients may be diagnosed than before. Consequently, orthopedists and hematologists face the imminent task of providing sufficient hemostasis while minimizing the risk of thrombotic events.

In our patient, the application scheme of TXA and LMWH is also worth discussing. Afibrinogenemia and hypofibrinogenemia patients who underwent total joint arthroplasty accepted a single or combined use of the 2 drugs.^[14–16]^ In terms of the aforementioned cases, TXA and LMWH seem to have achieved satisfactory outcomes (Table 2). The relationship between CD and complications following TKA is less clearly defined, although in many pregnancy cases, there is still no unified application of the aforementioned drugs.^[12]^ Furthermore, the coagulation balance in asymptomatic CD patients should be carefully considered for treatment with FC, TXA, and LMWH.

**Table 2:** Summary of afibrinogenemia and hypofibrinogenemia cases with arthroplasty.

Surgery	Diagnosis	Personal history	Family history	Routine treatment			Outcome
				FC	LMWH	TXA	
Total elbow arthroplasty^[14]^	Afibrinogenemia	Spontaneous bleeding	-	>1.0 g/L	Dosing	Dosing	Well
Total hip arthroplasty^[16]^	Afibrinogenemia	Spontaneous bleeding	-	>1.0 g/L	-	Dosing	Well
Total knee arthroplasty^[15]^	Hypofibrinogenemia	Negative	Bleeding	>1.0 g/L	Dosing	Dosing	Hematoma and infection

FC = fibrinogen concentrate, LMWH = low molecular weight heparin, TXA = tranexamic acid.

Because of the performance of asymptomatic CD patients after supplementation, we hypothesize a close similarity in structure and function between variants and fibrinogen. It requires more case-controlled trials to explore the safety and efficacy of low doses. As in all surgery, complications after TKA in those patients require close cooperation among multidisciplinary teams to prevent their occurrence.

## Author contributions

DC.M. and J.W. contributed to the case management, analyzed the data, and drafted the manuscript. RZ.Z., CN.J., and SJ.G. contributed significantly to perform the literature revision, and collected the data responsibility for the entire content of this submitted manuscript and approved submission.

**Resources:** Chenni Ji.

**Software:** Runzi Zhang.

**Validation:** Shijun Gao.

**Writing – original draft:** Decheng Meng, Juan Wang.

**Writing – review & editing:** Decheng Meng, Juan Wang.
